# The role of objective and subjective effort costs in voluntary task choice

**DOI:** 10.1007/s00426-021-01587-2

**Published:** 2021-08-29

**Authors:** Gesine Dreisbach, Vanessa Jurczyk

**Affiliations:** 1 Department of Psychology, University of Regensburg, Universitätsstraße 31, 93053 Regensburg, Germany; 2 Bremen University, Bremen, Germany

## Abstract

Human beings tend to avoid effort, if a less effortful option is equally rewarding. However, and in sharp contrast to this claim, we repeatedly found that (a subset of) participants deliberately choose the more difficult of two tasks in a voluntary task switching (VTS) paradigm even though avoidance of the difficult task was allowed (Jurczyk et al., Motivation Science 5:295–313, 2019). In this study, we investigate to what extent the deliberate switch to the difficult task is determined by the actual objective or the subjective effort costs for the difficult task. In two experiments, participants (*N* = 100, each) first went through several blocks of voluntary task choices between an easy and a difficult task. After that, they worked through an effort discounting paradigm, EDT, (Westbrook et al., PLoS One 8(7):e68210, 2013) that required participants to make a series of iterative choices between re-doing a difficult task block for a fixed amount or an easy task block for a variable (lower) amount of money until the individual indifference point was reached. In Experiment 1, the EDT comprised the same tasks from the VTS, in Experiment 2, EDT used another set of easy vs. difficult tasks. Results showed that the voluntary switch to the difficult task was mostly predicted by the objective performance costs and only marginally be the subjective effort cost. The switch to the difficult task may thus be less irrational than originally thought and at its avoidance at least partially driven by economic considerations.

**Supplementary Information:**

The online version contains supplementary material available at 10.1007/s00426-021-01587-2.

## Explaining human action selection

Human beings have a huge repertoire of potential behaviors they can select from at any given moment. And it is one of the core questions of psychology to find out what determines this choice. One aspect that has moved into the focus of research during the last decade is how the expected costs of an action modulate its selection (Berkman et al., 2017; Kurzban et al., 2013; Shenhav et al., 2013, 2021). According to the law of least effort (Hull, 1943), humans always strive for the least effortful (costly, that is) behavior. While originally restricted to physical effort, accumulating evidence suggests that this effort avoidance also holds for cognitive effort (Kool et al., 2010). However, the world is full of examples where people deliberately go for the more effortful choice even in leisure activities like mountain climbing or chess playing instead of taking the cable car or watching TV (Inzlicht et al., 2018). One potential reason for this effort paradox might be that people have different objective and subjective effort costs for the same task. That is, the trained mountain climber may in fact have to invest less physical effort (low *objective* costs) and the chess player may enjoy the cognitive effort of chess playing (low *subjective* costs). In this paper, we aim to investigate whether and how the objective and subjective costs for a given task may in part solve the effort paradox.

**Fig. 1:**
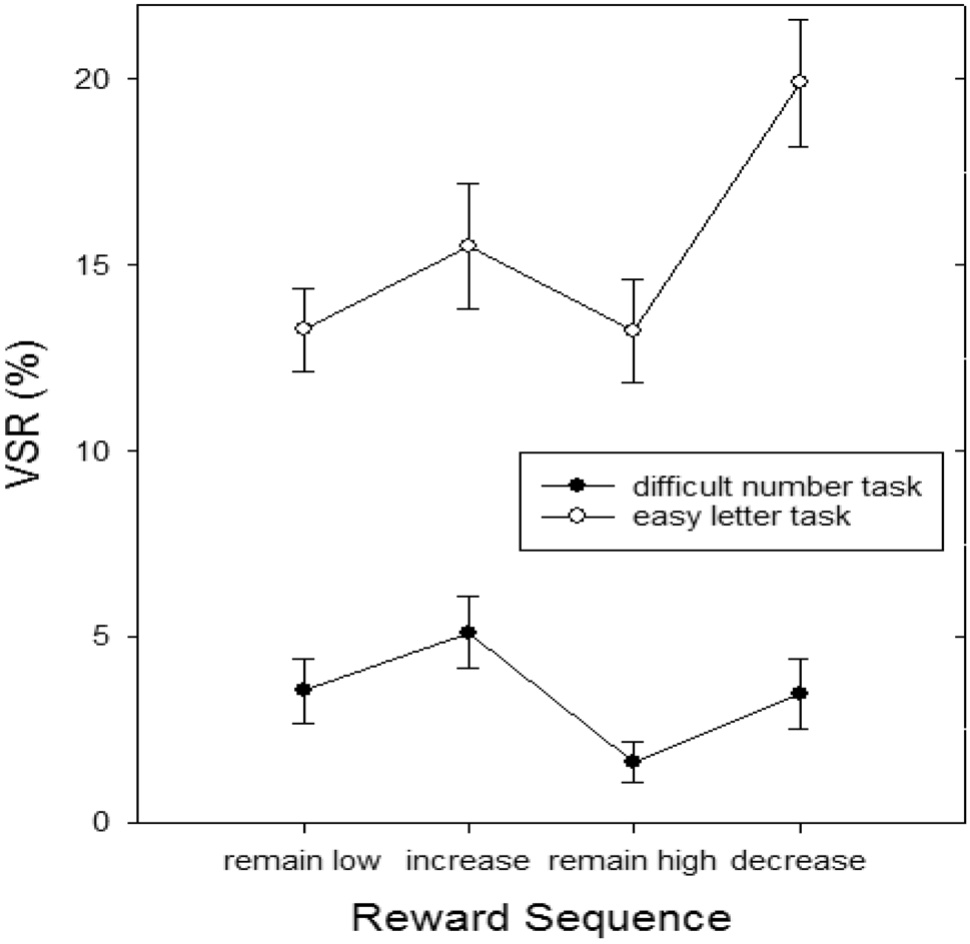
Voluntary switch rate (VSR) as a function of task type (easy/difficult) and reward sequence (from Jurczyk et al., 2019)

In the laboratory, the choice between different cognitive tasks can be investigated with the voluntary task switching paradigm (Arrington & Logan, 2004, 2005; Arrington et al., 2014). The typical finding in this paradigm is that participants strongly prefer to repeat the task and barely choose to switch unless the instructions tell them to do so at least on a certain subset of trials (e.g., (Kessler et al., 2009). This repetition bias (the strong preference for task repetitions) can be taken as another indication of effort avoidance (Kool et al., 2010), because participants avoid the switch costs that are typically associated with a task switch (Dreisbach, 2012; Kiesel et al., 2010; Monsell, 2003; Vandierendonck et al., 2010). That means, any truly voluntary task switch (truly in the sense that the instructions would allow participants to always repeat the task on free choices) contradicts the law of least effort. Interestingly, there is evidence that the voluntary switch rate can reliably be increased when specific reward conditions are met: Fröber and Dreisbach (2016) used cues that announced either a high or low reward randomly changing between trials. And they repeatedly found increased switch rates when reward prospect increased from one trial to the next (but not when reward remained high) even though the reward was provided contingent on fast and correct responding but *not* on the choice to switch (Fröber & Dreisbach, 2016; Fröber et al., 2018, 2019). What is even more, Jurczyk et al., (2019) showed that this sequential reward effect of higher VSR when reward prospect increased is also found when participants have to switch between an easy and a difficult task: while only few participants switched to the difficult task (when they could also repeat the easy task), those who did, did so especially when reward prospect increased (see Fig. 1).

Recently, it was argued that the general reward sequence effect of higher VSR when reward prospect increased (as compared to unchanged high reward) could be explained by a lowering of the updating threshold in working memory in response to the unexpected in increase in reward (Cools & D’Esposito, 2011; Dreisbach & Fröber, 2019). The assumed mechanism could be that a lower threshold goes along with a change in brain signal variability, thereby increasing the probability that a less activated task (like the one that was not just recently executed) is chosen (Faisal et al., 2008; Garrett et al., 2015; Waschke et al., 2021). However, to date we have not yet identified why a subset of participants voluntarily switches to the more difficult of two tasks (when reward prospect increases). One reason that we aim to investigate here might be that some participants experience the more difficult task not as effortful as others do. And this might be true either because for them, executing the difficult task is in fact less costly, or, simply because they do not mind the higher costs. Here, we will use the individual reaction time difference between the easy and the difficult task as a proxy for the actual objective performance costs. And we will use a cognitive effort discounting task (EDT) to measure the individual subjective effort costs for the difficult task as recently introduced by Westbrook and colleagues (Westbrook & Braver, 2015; Westbrook et al., 2013). Participants will first work through several task switching blocks using the hybrid task switching paradigm with 50% forced choices (half switch, half repetitions) and 50% free choices. The hybrid paradigm allows to abandon any task instruction that asks participants to switch at least on a subset of trials and instead leaves it entirely to the participant which task to choose on free choice trials (Fröber & Dreisbach, 2017). As in Jurczyk et al. (2019), we will use a difficult and an easy task and will present a cue that announces either a high or low reward contingent on performance (but not on choice). After this task switching phase, the EDT will be conducted. Participants will have to choose whether they would be willing to do another block only including the difficult task for 2€ or a block of the easy task for 1€. If the participant choses the difficult task, the amount for the easy task will be increased by 50 Cents, if they go for the easy task, it will be decreased by 50 Cents. This procedure will be repeated for five more offers, and for each subsequent offer, the reward available for the single task block will be adjusted by half as much as the previous adjustment (see “Methods”). The difference between the final offer for the easy task and the 2€ then determines the subjective effort costs for a given participant: it is the amount of money a participant is willing to forego to avoid the more difficult task. The main question of interest is whether and to what extent the objective performance costs (individual RT difference between difficult and easy task) and the subjective effort costs will predict the voluntary switch rate to the difficult task. Note that deciding to switch to the difficult task is only possible when the easy task was just executed. That means, on voluntary task switches to the difficult task, participants not only choose a more difficult task, they also choose the less activated task. It follows that switching to the difficult task should have the most effortful consequences.^1^ And aside from the overall VSR to the difficult task, we will also use the VSR to the difficult task specifically when reward prospect increases as criterion, because this is where we found the highest VSR in Jurczyk et al. (2019) and because this ought to be the costliest decision.

## Experiment 1

### Methods

#### Participants

The minimum sample size of a multiple regression with two predictors and a medium effect size of 0.15, a power of 0.95 and *α* = 0.05 would require 74 participants (Faul et al., 2009). To be on the save side, given the novelty of our approach, we decided upfront to collect data from 100 participants. Consequently, 100 students from the University of Regensburg participated in this study for course credit or money (6€). On top, they could earn up to 2€ in the last effort discounting block (description see below). Subjects were between 18 and 47 years old (*M* = 22.9 years; *SD* = 3.9). Of all participants, 41 studied Psychology, 84 were female and 7 were left-handed. All participants had normal color vision as confirmed by means of an Ishihara test. Informed consent was provided by all subjects prior to the experiment.

#### Apparatus

The experiment was run using E-Prime 2.0 (Psychology Software Tools, Sharpsburg, PA) on a 19-inch TFT-monitor (display resolution at 1280 * 1024 pixels, refresh rate 60 Hz). Responses were collected with a QWERTZ-keyboard, using “y” and “x” as the left and right response key for one task (left hand), and “n” and “m” as left and right response key for the other task (right hand). Participants were seated at approximately 60 cm from the screen (unconstrained). At this distance, 1 cm on screen corresponds to approximately 1° of visual angle.

#### Stimuli and procedure—task switching

On each trial, participants either responded to an easy letter task or a more difficult prime number task. In the relatively easier letter task, a letter stimulus (B, D, F, H, S, U, W, or Y) had to be categorized as being nearer to A vs. nearer to Z in the alphabet by pressing a left or right response key with the left or right hand, respectively. In the relatively more difficult number task, an appearing number (15, 17, 19, 21, 23, 25, 27, or 29) had to be categorized as prime number vs. not a prime number. The stimulus of one task always appeared above a central fixation dot (Origin font, 28 pt.) and the stimulus of the other task below (each presented with 1.5° distance to the fixation dot). Mapping of number or letter task to position on the screen was fixed but counterbalanced across participants, while responses to the upper task were always given with the left hand. All stimuli were displayed in black ink (Calibri font, bold, 28 pt., bold, ~ 1° of visual angle) on a white background—and their number and identity indicated trial type and task, respectively: When two stimuli appeared on a given trial, participants were free to choose which task to perform (voluntary task choice), while a single stimulus indicated a forced-choice trial. Note that instructions emphasized that participants were completely free in their decision—in contrast to for example the standard voluntary task switching instruction introduced by (Arrington & Logan, 2004).

The experiment started with two short single-task practice blocks comprising 16 trials each, so that each stimulus randomly appeared 2 times, always starting with the difficult prime number task. This was followed by one practice block of forced-choice trials (half switch, half repeat trials), and another of voluntary task switching trials (16 trials each). A subsequent baseline block of 128 trials consisted of both forced-choice and free-choice trials (half forced, half free, pseudorandomized with stimulus repetitions excluded and 50% switches on the forced-choice trials). During this baseline block, individual RT thresholds for earning the high reward in the following test phase were calculated as the 30th percentile (correct RTs ordered from fast to slow) separately for each condition. The following test phase consisted of two blocks of 256 trials each. All stimuli appeared equally often, but without direct stimulus repetitions, pseudorandomized so that all conditions (Reward sequence × Trial type × Task) appeared about equally often and equally distributed.^2^ Participants were encouraged to rest between blocks.

**Fig. 2:**
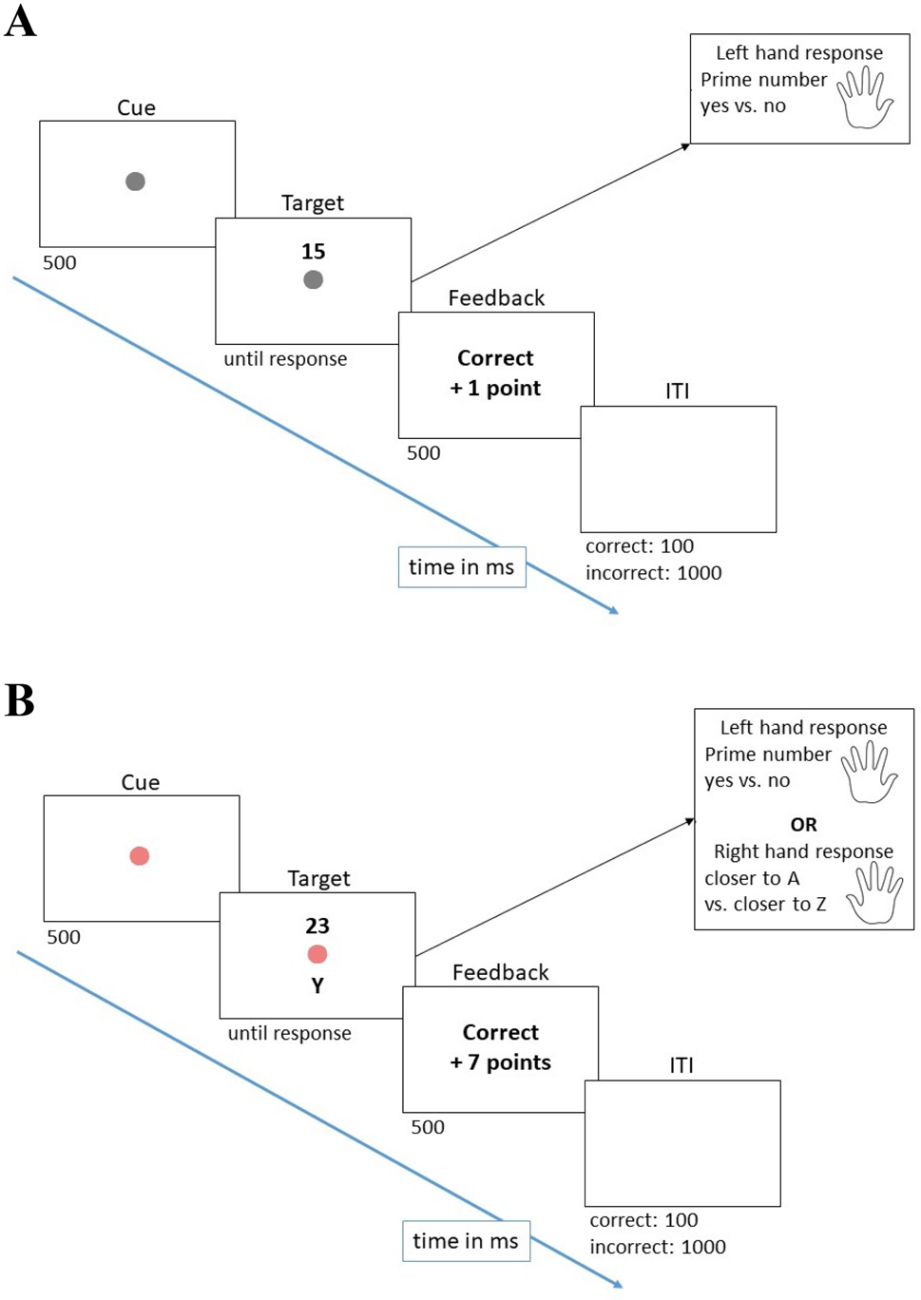
Trial procedure of a forced-choice trial (**A**) and a free-choice trials (**B**)

The trial procedure for both forced-choice and free-choice trials is depicted in Fig. 2. Each trial began with the presentation of the reward cue. To allow for changes of the reward cue on every trial, we used three different colors per reward magnitude: low reward was always announced by one of three different hues of gray (RBG values: 169, 169, 169; 128, 128, 128; 90, 90, 90). High reward was announced by a task cue displayed in either blue (30,144, 255), red (240, 128, 128), or yellow (255, 215, 0). On low reward trials, each correct response was rewarded with one point, on high reward trials participants could gain seven points for correct responses below the individual RT criterion from the baseline block. After 500 ms, the stimulus (on forced-choice trials) or stimuli (on free-choice trials) appeared on screen until a response was given. A feedback display was presented for 500 ms, informing the participants about whether they earned the reward on a given trial. If they made an incorrect response, the German word for wrong (“Falsch”) appeared on the screen and, in case of a high reward prospect, a correct, but too slow response was followed by a feedback screen where the words “Zu langsam” (too slow) were shown. In case of a correct (and for high-reward prospect trials, fast enough) response, “Richtig” (correct) was displayed. This was followed by either a short (100 ms) intertrial interval (ITI) after correct responses or a long (1000 ms) ITI after an error was made.

At the beginning of the test phase, participants were told that they could earn points for correct (low reward) or fast and correct responses (high reward) to take part in a competition: the best three participants in the experiment won a 15, 10, and 5 € Amazon voucher (cf. Fröber et al., 2018).

#### Effort discounting task

**Fig. 3:**
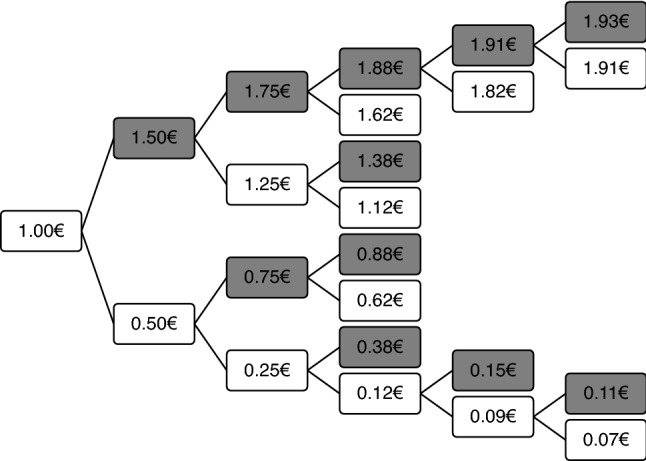
EDT procedure in Experiments 1 and 2. The offer for the difficult task-block remains fixed at 2 Euros on every choice. The offer for the easy task-block shown here starts with 1 Euro and is then sequentially adjusted depending on the participant’s individual choice (increase when the difficult task is chosen, decrease when the easy task is chosen). The grey offers are the ones following a decision for the difficult task (i.e., increase the offer for the easy task), the white offers follow a decision for the easy task. Not all potential outcomes are shown in the figure

In the effort discounting procedure adapted from Westbrook et al. (2013), participants were told that they had to perform another block of either the letter or the digit task. They could decide between either doing another block of the digit task for a fixed amount of 2 Euros, or the letter task for a smaller amount. Before this last block of 32 single-task trials, participants had to go through 6 effort discounting trials: in the first, they were offered the choice of either doing a difficult-taskblock for 2€ or an easy-task block for 1€.^3^ Depending on their choice, the amount offered for the easy-task block was adapted in a trial-wise staircase procedure: it was incrementally increased or decreased by 50cts, 25cts, 13cts, depending on whether the participants chose to do the difficult task or not. An example of such a decision tree is depicted in Fig. 3. To emphasize the seriousness of the respective decisions, participants were told that one of their decisions would be randomly chosen as the task type and amount of money for the last block (while in fact, the decision of the third effort discounting trial was used for the last block). They were also instructed that the monetary offer would only be paid if performance on this last block was sufficiently fast and accurate.

#### Design

The voluntary switch rate on free choice trials was the main dependent measure in a two (task difficulty: easy, difficult) × 4 (reward sequence: remain low, increase, remain high, decrease) repeated-measures design. The VSR was defined as the number of switches to a task in a given reward-sequence condition relative to the number of all free-choice trials in that condition. In addition, we analyzed reaction times (RT, in ms) and error rates (ERR) as a function of task difficulty, task transition (repeat vs. switch), and reward sequence on forced-choice trials.^4^ We calculated the objective effort costs as the overall difference between RTs for (forced) difficult task and the (forced) easy task. We calculated the subjective value of effort for each participant as the difference between the amount of money offered for an easy-task block and the fixed 2 Euros for the difficult-task block in the last effort discounting trial: that is, if participants had a high difference between those amounts (a high subjective value of effort), participants would forgo a higher amount of money to avoid the more difficult task. Subjective and objective effort costs will be used as predictors and the overall VSR to the difficult task and VSR to the difficult task when reward prospect increases as criterion in a multiple linear regression.

### Results

#### Data preprocessing

Before all data analyses, the first trial of each block was excluded (0.4% of all trials), as it does not entail task transition nor reward sequence. Four subjects^5^ were excluded, either due to extremely high ERRs in the difficult task (with an accuracy that was not considerably different from chance, three subjects) or due to extremely high ERRs in the easy task (deviating more than three interquartile ranges from the grand median, one subject). For the RT analysis only, we further excluded incorrect trials (12.9%), trials immediately following incorrect responses (11%) and trials with RTs that were three *SD*s above or below the subject’s cell mean (0.74%). In the VSR analysis, correct and erroneous trials were included to cover all attempts of deliberate switching (Arrington & Logan, 2004). In error trials, the chosen task was inferred from the selected hand as it is assumed that participants rather choose the wrong finger than the wrong hand (Scheffers & Coles, 2000).

#### VSR analysis

**Fig. 4:**
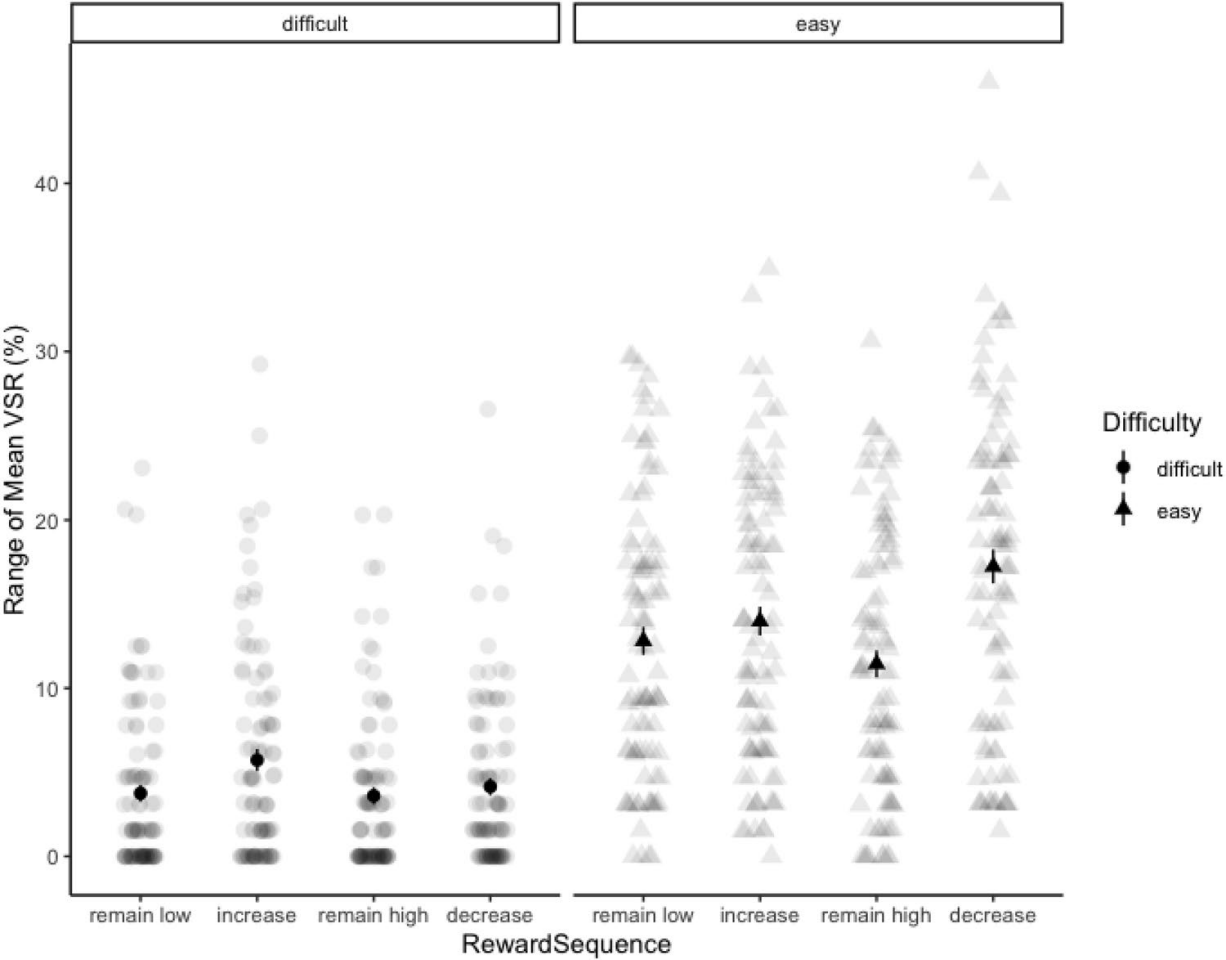
Range of individual mean voluntary switch rates in Experiment 1 as a function of task difficulty and reward sequence. Block dots represent respective overall means (± 1 SEM)

Individual and overall mean VSR are depicted in Fig. 4. The two main effects and the interaction were highly significant: participants chose the easy task more often than the difficult task, *F*(1, 95) = 75.40, *p* < 0.001, ${\eta}_{p}^{2}$ = 0.44, they switched more often when reward prospect increased*, F*(3, 285) = 28.78, *p* < 0.001, ${\eta}_{p}^{2}$ = 0.23, and reward sequence had a stronger effect on choices for the easy task, *F*(3, 285) = 14.41, *p* < 0.001, ${\eta}_{p}^{2}$ = 0.132. Critically, and replicating the findings from Jurczyk et al., (2019), participants chose the difficult task more often when reward prospect increased compared to all other reward sequences: increase vs. remain low, *F*(1, 95) = 19.08, *p* < 0.001, ${\eta}_{p}^{2}$ = 0.17, increase vs. remain high, *F*(1, 95) = 19.34, *p* < 0.001, ${\eta}_{p}^{2}$ = 0.17, and increase vs. decrease, *F*(1, 95) = 8.44, *p* = 0.005, ${\eta}_{p}^{2}$ = 0.08. All other comparisons were nonsignificant (all *F*s < 2.10, all *p*s > 0.15). For the easy task, the picture was more complex, as all reward sequences significantly deviated from one another (all *F*s > 5.70, all *p*s < 0.020), except for the only marginally significant difference between remain low and increase, *F*(1, 95) = 3.19, *p* = 0.077. The highest VSR to the easy task could be found under decreasing reward prospect, followed in descending order by the increase, remain low, and remain high condition.

#### Forced-choice trials, RT data

In the RT analysis, all three main effects were significant: participants were slower on the difficult as compared to the easy task, *F*(1, 95) = 216.3, *p* < 0.001, ${\eta}_{p}^{2}$ = 0.70. They were slower on task switches as compared to task repetitions, *F*(1, 95) = 245.3, *p* < 0.001, ${\eta}_{p}^{2}$ = 0.72, and they were generally faster on high reward (increase, remain high) as compared to low reward (decrease, remain low) trials, *F*(3, 285) = 17.4, *p* < 0.001, ${\eta}_{p}^{2}$ = 0.16. These main effects were qualified by three two-way interactions: switch costs were larger for the more difficult task than for the easy task (156 ms vs. 99 ms), as indicated by the interaction of task and task transition, *F*(1, 95) = 27.0, *p* < 0.001, ${\eta}_{p}^{2}$ = 0.22. Moreover, the difference in RTs between high and low reward prospect was more pronounced for the difficult task, task × reward sequence, *F*(3, 285) = 4.27, *p* = 0.006, ${\eta}_{p}^{2}$ = 0.04. In the high-reward conditions (increase and remain high) as compared to the low-reward conditions (decrease and remain low), participants were 62 ms faster in the difficult task, but only 30 ms faster in the easy task. Finally, the interaction of transition and reward sequence was significant, *F*(3, 285) = 7.60, *p* < 0.001, ${\eta}_{p}^{2}$ = 0.07. Irrespective of task difficulty, repetitions were fastest with remaining high reward prospect (all post hoc comparisons significant, all *F*s > 4.39, all *p*s < 0.040), and switches were fastest in the increase condition (all *F*s > 5.90, all *p*s < 0.018), replicating previous findings (Fröber & Dreisbach, 2016; Shen & Chun, 2011). The three-way interaction was not significant (*F* < 0.40, *p* > 0.84). Descriptive statistics are provided in Table 1. Finally, we computed the individual mean difference RT between easy and difficult task (*M*_difficult_ – *M*_easy_) on forced choices which was later entered as predictor in the multiple regression, the overall mean difference was 192 ms (range − 89.85–515.47 ms).

**Table 1:** Mean RTs (ms) and error rates (%) for the easy and difficult task as a function of reward sequence and task transition on forced choice trials in Experiment 1

	Remain low		Increase		Remain high		Decrease	
	Repetition	Switch	Repetition	Switch	Repetition	Switch	Repetition	Switch
	Difficult task							
RT (*SD*)	696 (*142*)	878 (*250*)	674 (*152*)	803 (*190*)	645 (*150*)	816 (*207*)	735 (*237*)	876 (*243*)
ERR (*SD*)	17.9 (*15.6*)	20.6 (*14.3*)	20.3 (*16.2*)	22.3 (*14.1*)	18.9 (*13.7*)	25.0 (*16.0*)	18.5 (*11.7*)	24.9 (*16.0*)
	Easy task							
RT (*SD*)	533 (*89*)	648 (*144*)	519 (*82*)	597 (*111*)	499 (*81*)	618 (*118*)	543 (*100*)	628 (*138*)
ERR (*SD*)	9.46 (*7.89*)	9.07 (*8.86*)	10.5 (*8.42*)	10.6 (*12.2*)	9.55 (*8.25*)	11.0 (*9.55*)	8.01 (*7.77*)	8.06 (*10.1*)

##### Forced-choice trials, ERR data

The same analysis on the ERR data revealed main effects of task, *F*(1, 95) = 189.8, *p* < 0.001, ${\eta}_{p}^{2}$ = 0.67, transition, *F*(1, 95) = 21.3, *p* < 0.001, ${\eta}_{p}^{2}$ = 0.18, and reward sequence, *F*(3, 285) = 2.67, *p* = 0.048, ${\eta}_{p}^{2}$ = 0.03. Participants made more errors on the more difficult task (21.0% vs. 9.5%), on task switches vs. task repetitions (16.4% vs. 14.1%), and on high reward trials as compared to low reward trials (16.0% vs. 14.5%). In addition, both two-way interactions involving the factor task were significant: task × transition, *F*(1, 95) = 16.8, *p* < 0.001; ${\eta}_{{p}}^{2}$ = 0.15 (switch costs of 4.3% vs. 0.3%, for the difficult and easy task, respectively), and of task × reward sequence, *F*(3, 285) = 2.64; *p* < 0.001; ${\eta}_{{p}}^{2}$ = 0.03. In both tasks, participants made more errors in high-reward trials compared to remain-low trials; in decrease trials, though, error rates were lowest in the easy task, but comparable to high-reward trials in the difficult task (see also Table 1). All other effects were nonsignificant (all *F*s < 2.20, all *p*s > 0.103).

#### Multiple linear regression

To test our main hypothesis of whether the higher voluntary switch rate to the difficult task could be predicted by the subjective effort costs for the difficult task and/or the actual RT costs for the difficult task, we calculated the subjective individual effort costs as the difference between the final amount of money for the easy task and the 2 Euro for the difficult task. Moreover, the mean RT difference of forced choice trials between the difficult and the easy task for each individual was taken as a marker for the actual effort costs.^6^ Both, subjective and objective effort costs were then entered as predictors into two multiple regressions with the overall VSR to the difficult task (irrespective of reward prospect) and with the VSR to the difficult task when reward prospect increased as dependent measure.

##### Overall VSR to the difficult task as DV

The regression equation was significant, *F*(2, 95) = 31.88, *p* < 0.001. The *R*^2^ for the overall model was 0.41 (adjusted *R*^2^ = 0.39), indicative for a high goodness-of-fit according to Cohen (1988). Participants’ predicted VSR to the difficult task is 8.80–0.022 (RT costs)—0.39 (subjective effort costs). However, only the actual RT costs were a significant predictor of the VSR, *β* = − 0.616, *t*(95) = − 7.08, *p* < 0.001, whereas subjective effort cost was not, *β* = − 0.05,* t*(95) = − 0.57, *p* = 0.57. The negative beta weights show that individual VSR to the difficult task decreased with increasing performance costs.

##### VSR to the difficult task when reward prospect increases as DV

**Fig. 5:**
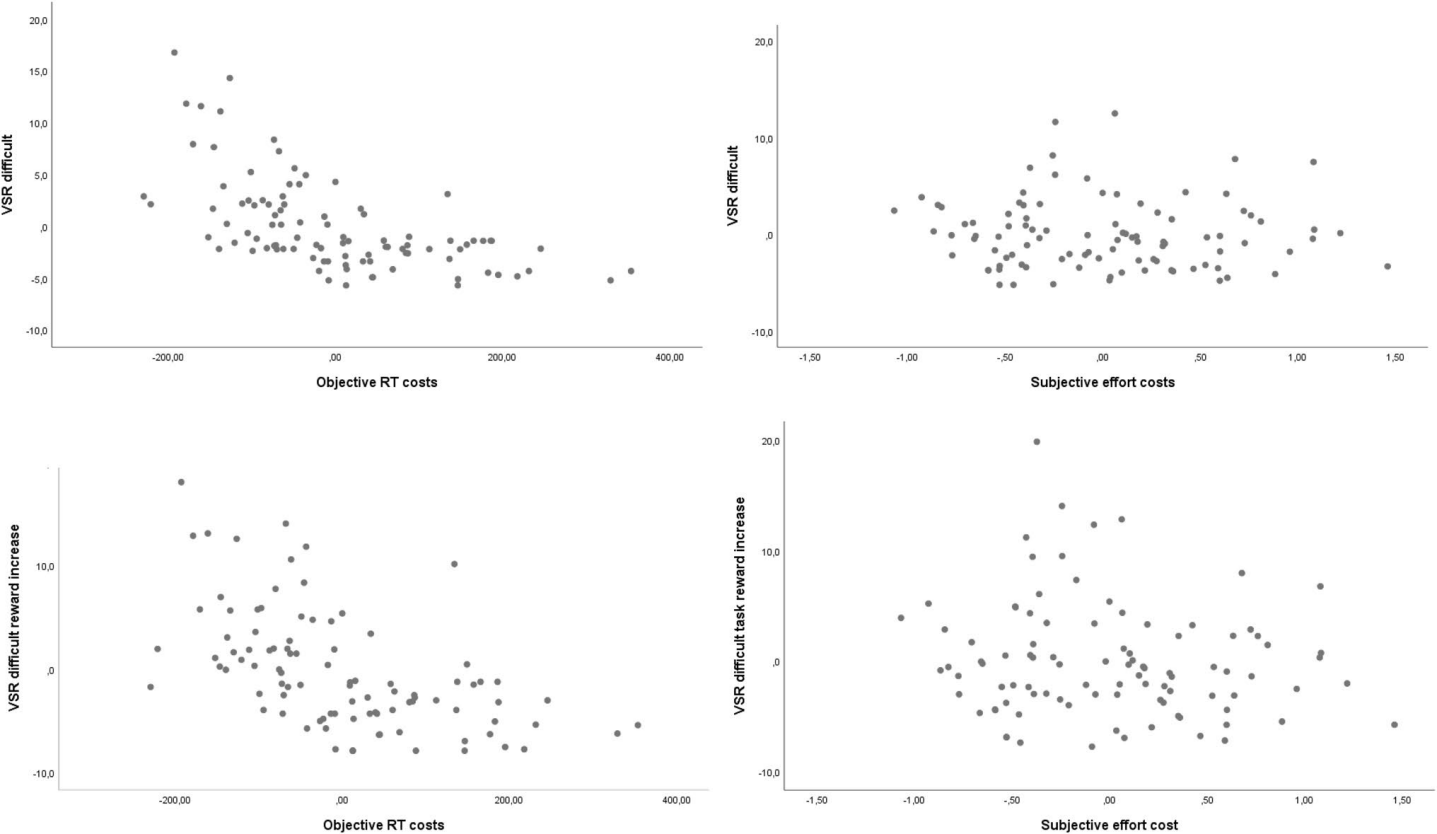
Partial regression plots (Experiment 1) of VSR to the difficult task (upper panels) of VSR to the difficult task when reward prospect increased (lower panels) vs. objective effort costs (left panels), and vs. subjective effort costs (right panels). *VSR* voluntary switch rate

Again, the regression equation was significant, *F*(2,95) = 26.48, *p* < 0.001. *R*^2^ for the overall model was 0.36 (adjusted *R*^2^ = 0.35), indicative for a high goodness-of-fit (Cohen, 1988). Participants’ predicted VSR to the difficult task when reward prospect increases is 11.69–0.028 (RT costs) – 1.23 (subjective effort costs). However, only the actual RT costs were a significant predictor of the VSR to the difficult task, *β* = − 0.55, *t*(95) = − 6.06, *p* < 0.001, whereas subjective effort cost was not, *β* = − 0.115, *t*(95) = − 1.27, *p* = 0.20. The negative beta weights show that individual VSR to the difficult task on reward increase trials decreased with increasing performance costs. Figure 5 shows the corresponding partial regression plots. Finally, we conducted a sensitivity analysis for multiple linear regression using GPower (*α* = 0.01, power = 0.95, *N* = 96, 2 predictors) which resulted in a minimum effect size of 0.23 and a critical *F*-value of 4.84 allowing to interpret the significant effects of our analyses as true positives (Faul et al., 2009).

### Discussion

We replicated the VSR findings from Jurczyk et al. (2019) and again found that participants generally switched more often when reward prospect increased as compared to unchanged high reward. Critically, this effect was again also found for the difficult task: even though the VSR to the difficult task was lower, a subset of participants voluntarily chose the more difficult task especially when reward prospect increased. This allowed to look into potential sources of this seemingly irrational behavior. Two individual markers, the subjective effort costs for the difficult task and the objective RT costs for the difficult task were used to predict the overall VSR to the difficult task and more specifically when reward prospect increased. Results from linear regression analyses show that our predictors explained between 35 and 39% of the variance. A closer look at the beta weights shows that the objective RT costs explained most of the variance: the higher the objective RT costs for the difficult task, the less a participant switched voluntarily to the difficult task. The subjective effort costs did not further predict VSR to the difficult task. This suggests that participants’ choice of the difficult task was modulated by the ease with which they were able to accomplish the task and not by the subjective effort costs. However, in Experiment 1, the objective and subjective costs were measured for the exact same set of tasks. Therefore, participants may have used introspective RT costs for the difficult task to make their decision in the EDT (Bratzke & Bryce, 2019). In Experiment 2, we therefore aimed to measure objective costs and subjective effort costs independently from each other. To do so, we re-ran the same hybrid voluntary task switching experiment, but used different tasks in the EDT in Experiment 2. In particular, we added two EDT blocks, one with data-limited tasks and one with resource-limited tasks (Norman & Bobrow, 1975). Data-limited means that increasing effort does not help to improve performance as for example in perceptual fluency tasks (Westbrook & Braver, 2015). Since low perceptual fluency is experienced as aversive and therefore is to be avoided (Dreisbach & Fischer, 2011; Reber et al., 1998; Song & Schwarz, 2008), the effort costs in this task is suited to measure the subjective aversion costs separately from the effort avoidance costs. Resource-limited means that increasing effort *does* improve your performance, like for example in math tasks. To sum up, Experiment 2 comprised a hybrid task switching phase with voluntary and forced task choices between an easy and a difficult task and randomly changing reward prospects. This part was identical to Experiment 1. This was followed by two EDT blocks (counterbalanced across participants). In one EDT block, participants made iterative choices between an easy and a more difficult math task. This allowed to measure general subjective effort costs independent from the tasks participants already knew from the preceding task switching experience. In the other EDT block, participants made iterative choices between an easy and a more difficult fluency task. This allowed to measure the general costs of aversive tasks (subjective aversion cost, hereafter).

## Experiment 2

### Methods

#### Participants

Another 100 students from the University of Regensburg participated in this second experiment. They were between 18 and 50 years old (*M* = 23.3 years; *SD* = 3.9). Of all participants, 37 studied psychology, 76 were female, and 3 were left-handed. Informed consent was provided by all subjects prior to the experiment.

#### Stimuli, procedure, and design

The entire task switching part was identical to Experiment 1. Instead of an effort discounting procedure using the same two tasks as in the test session, participants now worked through two effort discounting procedures involving different tasks (order counterbalanced). In the effort discounting task with math problems, participants made iterative choices between doing a block of non-carrying addition problems (easy task) or division problems in the multiplication table of the integers 3 through 9. In the effort discounting task with a fluency manipulation (adapted from Dreisbach & Fischer, 2011), participants could choose between a high fluency and easy-to-read (Arial font, 17 pt., black) or disfluent and more difficult-to-read (Mistral font, 22 pt, light grey, see Table 2 for examples for both tasks) number categorization block (categorizing single-digit numbers as being smaller or larger than five). Again, participants were told that they would have to carry out one block depending on one of their (randomly drawn) decisions, and would get the associated offered reward if they responded sufficiently fast and accurate. The math and the fluency tasks were chosen to make sure that participants would understand with the first encounter of the example we gave them (see Table 2), what the more difficult and what the easy task would be.

**Table 2:** Examples for the fluency and math tasks in the effort discounting procedure of Experiment 2

	Easy	Difficult
Fluency-task (subjective aversion costs)		
Math-task (cognitive effort costs)	2 + 5	36:9

Accordingly, we calculated the subjective aversion costs and subjective cognitive effort costs as the difference between the last offered amounts for the easy and the fixed 2 Euros for the difficult option in both EDT blocks.

### Results

#### Data preprocessing

Again prior to all analyses, the first trial of each block was excluded (0.4% of all trials). Before the RT analysis only, incorrect trials (13.2%), trials following errors (11.3%), and trials whose RTs deviated more than three *SD*s from the subject’s cell mean (0.8%). In addition, data of four participants were excluded from all analyses: two of them had a mean RT that deviated more than three interquartile ranges from the grand median and another two subjects made more than 50% errors in the difficult task.

#### VSR analysis

**Fig. 6:**
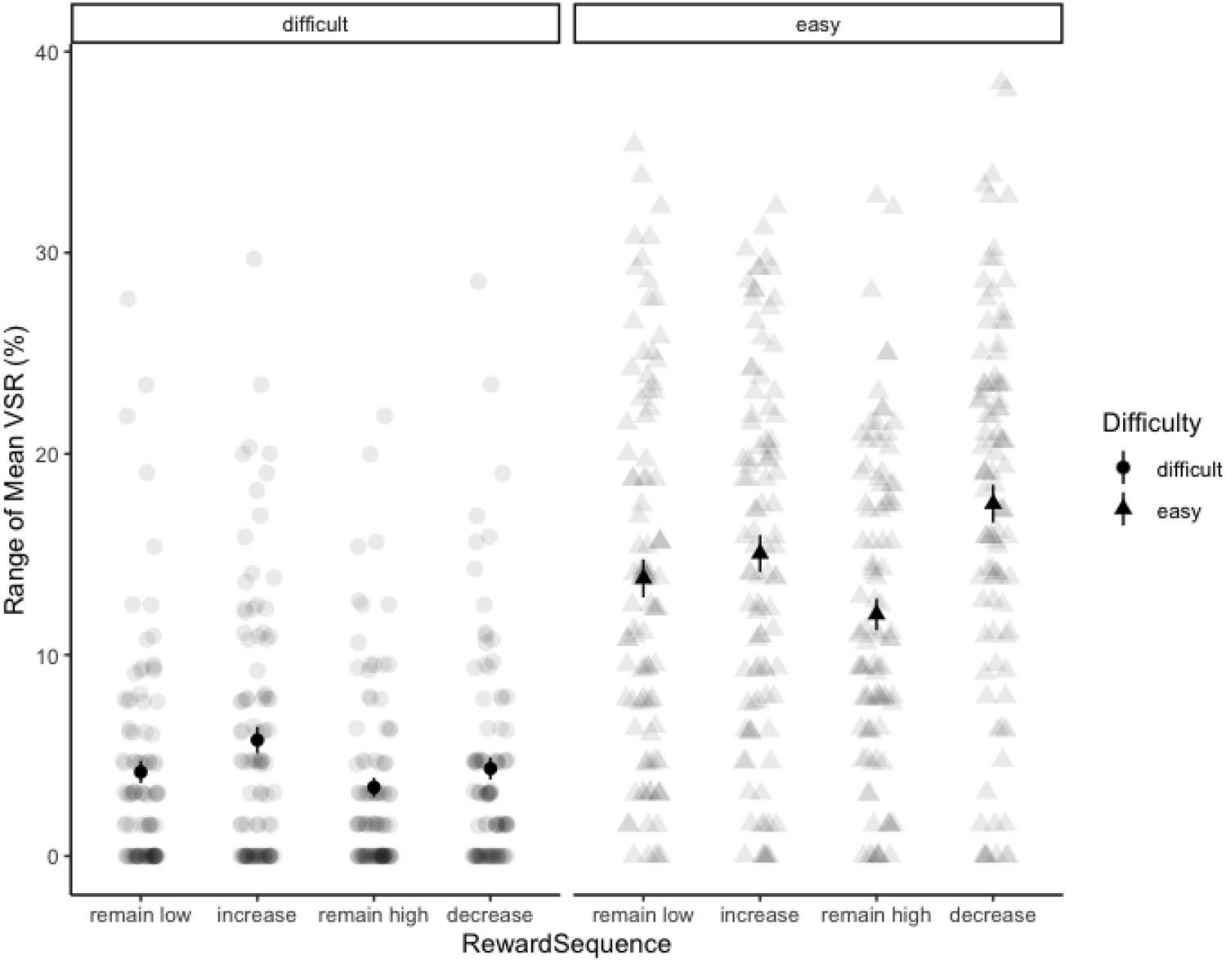
Range of individual mean voluntary switch rates in Experiment 2 as a function of task difficulty and reward sequence. Block dots represent respective overall means (± 1 SEM)

Individual and overall mean VSR as a function of reward sequence and difficulty are depicted in Fig. 6. As in Experiment 1, the main effects and the interaction were highly significant, main effect of task, *F*(1, 95) = 80.59, *p* < 0.001, ${\eta}_{p}^{2}$ = 0.46, main effect of reward sequence, *F*(3, 285) = 27.94, *p* < 0.001, ${\eta}_{p}^{2}$ = 0.23, and the interaction of task and reward sequence, *F*(3, 285) = 10.04, *p* < 0.001, ${\eta}_{p}^{2}$ = 0.10. Replicating Experiment 1, all planned comparisons regarding the VSR to the difficult task between increase and the other three reward sequence conditions were significant, with the highest VSR to the difficult task with increasing reward prospect: increase vs. remain low, *F*(1, 95) = 12.23, *p* < 0.001, ${\eta}_{p}^{2}$ = 0.11, increase vs. remain high, *F*(1, 95) = 25.03, *p* < 0.001, ${\eta}_{p}^{2}$ = 0.21, and increase vs. decrease, *F*(1, 95) = 6.92, *p* = 0.010, ${\eta}_{p}^{2}$ = 0.06. In addition, the VSR to the difficult task was lowest under remaining high reward prospect: remain high vs. remain low, *F*(1, 95) = 4.21, *p* = 0.043, ${\eta}_{p}^{2}$ = 0.04 remain high vs. decrease, *F*(1, 95) = 8.33, *p* = 0.005, ${\eta}_{p}^{2}$ = 0.08. In contrast, the VSR to the easy task was highest under *decreasing* reward prospect (and again lowest under remaining high reward prospect): decrease vs. remain low, *F*(1, 95) = 20.24, *p* < 0.001, ${\eta}_{p}^{2}$ = 0.18, decrease vs. increase, *F*(1, 95) = 8.57, *p* = 0.004, ${\eta}_{p}^{2}$ = 0.08, decrease vs. remain high, *F*(1, 95) = 57.97, *p* < 0.001, ${\eta}_{p}^{2}$ = 0.38, remain high vs. remain low, *F*(1, 95) = 10.23, *p* = 0.002, ${\eta}_{p}^{2}$ = 0.10, remain high vs. increase, *F*(1, 95) = 18.65, *p* < 0.001, ${\eta}_{p}^{2}$ = 0.16. All other single comparisons were nonsignificant (all *F*s < 3.14, all *p*s > 0.07).

#### Forced-choice trials, RT data

Again, all three main effects were significant, task, *F*(1, 93) = 188.2, *p* < 0.001, ${\eta}_{p}^{2}$ = 0.67, task transition, *F*(1, 93) = 276.2, *p* < 0.001, ${\eta}_{p}^{2}$ = 0.75, and reward sequence, *F*(3, 279) = 15.7, *p* < 0.001, ${\eta}_{p}^{2}$ = 0.14. The significant two-way interaction of task and transition replicates the finding of larger switch costs for the more difficult task as compared to the easy task (134 vs. 103 ms), *F*(1, 93) = 5.83, *p* = 0.018, ${\eta}_{p}^{2}$ = 0.06. Also, the interaction of transition and reward sequence was significant, *F*(3, 279) = 9.80, *p* < 0.001, ${\eta}_{p}^{2}$ = 0.10, with fastest repetitions in remain-high trials and fastest switches in increase trials. Neither the two-way interaction task × reward sequence, *F*(3, 279) = 2.20, *p* = 0.094, nor the three-way interaction, *F*(3, 279) = 1.5, *p* = 0.220, reached significance (for descriptive statistics see Table 3). We again computed the individual mean difference RT between easy and difficult task (*M*_difficult_ – *M*_easy_) on forced choices which was later entered as predictor in the multiple regression, the overall mean difference was 194 ms (range − 44.60–724.93 ms).

**Table 3:** Mean RTs (ms) and error rates (%) for the easy and difficult task as a function of reward sequence and task transition on forced choice trials in Experiment 2

	Remain low		Increase		Remain high		Decrease	
	Repetition	Switch	Repetition	Switch	Repetition	Switch	Repetition	Switch
	Difficult task							
RT (*SD*)	709 (*172*)	884 (*253*)	702 (*207*)	778 (*172*)	653 (*158*)	803 (*178*)	748 (*222*)	884 (*292*)
ERR (*SD*)	14.9 (*11.6*)	20.2 (*14.5*)	20.7 (*17.7*)	24.7 (*14.7*)	21.0 (*14.6*)	23.6 (*14.9*)	19.5 (*13.0*)	22.3 (*15.2*)
	Easy task							
RT (*SD*)	540 (*101*)	667 (*138*)	511 (*65.5*)	594 (*103*)	501 (*65.5*)	615 (*125*)	566 (*153*)	654 (*159*)
ERR (*SD*)	9.06 (*7.38*)	9.78 (*10.8*)	10.4 (*8.59*)	10.7 (*10.2*)	10.9 (*8.35*)	12.7 (*11.3*)	8.52 (*7.40*)	10.0 (*10.5*)

#### Forced-choice trials, ERR data

The ERR ANOVA replicated the results of Experiment 1. The three main effects were significant, task, *F*(1, 95) = 129.8, *p* < 0.001, ${\eta}_{p}^{2}$ = 0.58, transition, *F*(1, 95) = 15.4, *p* < 0.001, ${\eta}_{p}^{2}$ = 0.14, and reward sequence, *F*(3, 285) = 9.31, *p* < 0.001, ${\eta}_{p}^{2}$ = 0.09. Participants made more errors on the more difficult task vs. the easy task (20.8% vs. 10.3%), when they switched tasks as compared to repeating them (16.7% vs. 14.4%), and on high-reward trials in contrast to low-reward trials (16.8% vs. 14.3%). These main effects were qualified by two two-way interactions, task × transition, *F*(1, 95) = 6.18, *p* = 0.015; ${\eta}_{{p}}^{2}$ = 0.06 (switch costs of 3.7% vs. 1.1%, for the difficult and easy task, respectively), and of task × reward sequence, *F*(3, 285) = 3.70; *p* = 0.012; ${\eta}_{{p}}^{2}$ = 0.04. As in Experiment 1, switch costs were higher for the more difficult task (3.7% vs. 1.1%). And again in both tasks, participants made less errors in low-reward trials compared to high-reward trials; this difference was less pronounced for decrease trials of the difficult task and increase trials of the easy task. No other effect was significant (all *F*s < 0.90, all *p*s > 0.520; descriptive statistics in Table 3).

#### Multiple linear regression

To investigate whether the higher voluntary switch rate to the difficult task (when reward prospect increases) would again be predicted by the actual RT costs and whether and to what extent subjective effort costs and/or subjective aversion costs would further explain the variance, the following indices were used as predictors: we again calculated the subjective cognitive effort costs as the difference between the final amount of money for the easy addition task and the 2 Euros for the difficult division task. We further calculated the subjective aversion costs as the difference between the final amount of money for the fluent task and the 2 Euros for the disfluent task. Finally, the mean RT difference of forced choice trials between the difficult and the easy task for each individual was taken as a marker for the actual effort costs.^7^ All three, subjective cognitive effort, subjective aversion and objective effort costs were then entered as predictors into two multiple regressions with the overall VSR to the difficult task (irrespective of reward prospect) and with the VSR to the difficult task when reward prospect increased as dependent measure.

##### Overall VSR to the difficult task as DV

**Fig. 7:**
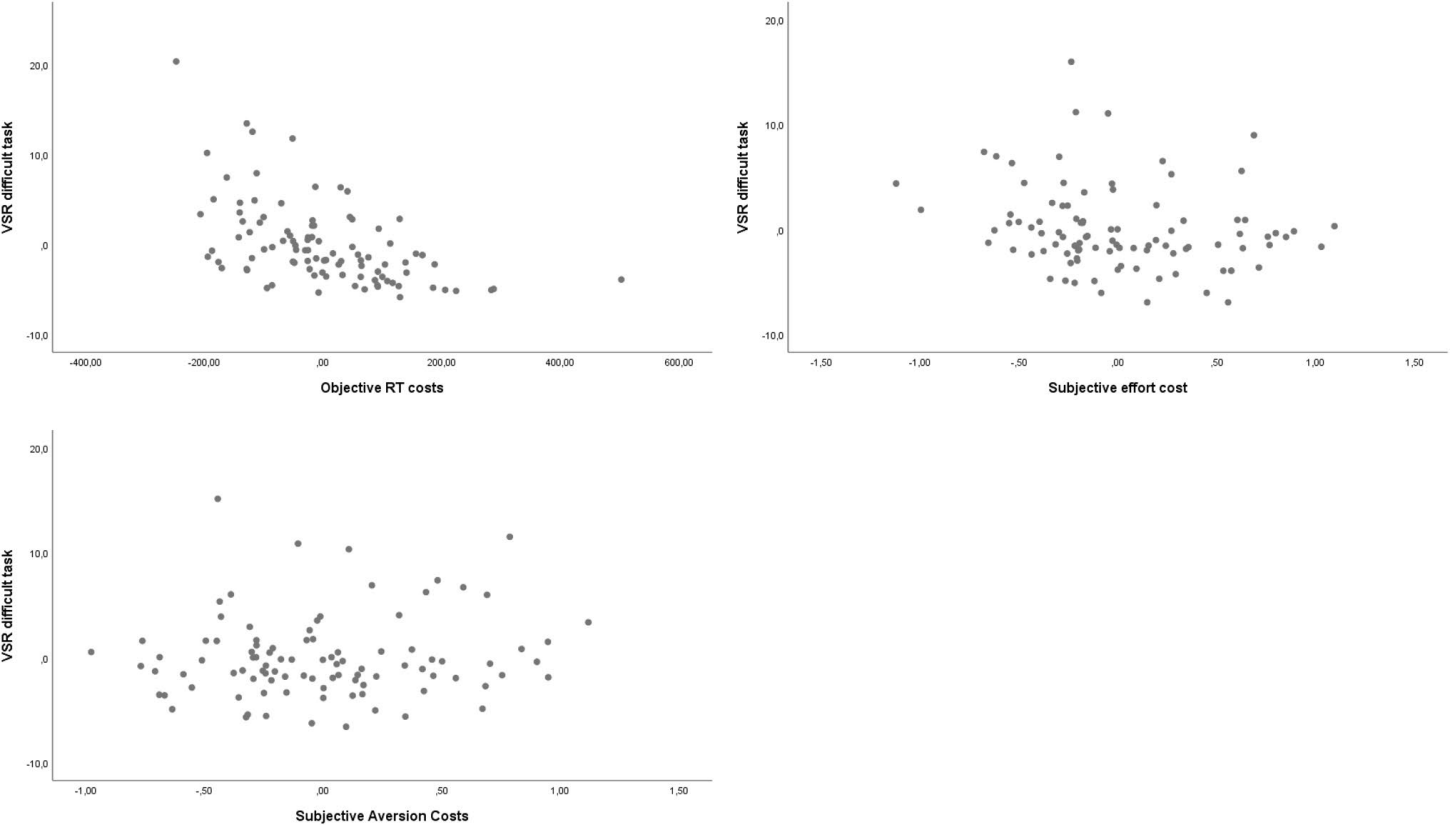
Partial regression plots (Experiment 2) of overall VSR to the difficult task vs. objective effort costs (upper left panel), vs. subjective effort costs (right panel), and vs. subjective aversion costs (lower panel). *VSR* voluntary switch rate

The regression equation was significant, *F*(2, 95) = 17.03, *p* < 0.001. The *R*^2^ for the overall model was 0.36 (adjusted *R*^2^ = 0.34), indicative for a high goodness-of-fit (Cohen, 1988). Participants’ predicted VSR to the difficult task is 9.11–0.019 (RT costs) – 1.82 (subjective cognitive effort costs) + 0.17 (subjective aversion costs). As in Experiment 1, the actual RT costs were again a significant predictor of the VSR to the difficult task, *β* = − 0.54, *t*(95) =  − 5.93, *p* < 0.001. This time, also the subjective effort costs seems to contribute significantly, *β* = − 0.20, *t*(95) = − 1.99, *p* = 0.050, whereas subjective aversion costs did not predict VSR to the difficult task, *β* = 0.11,* p* = 0.31. The negative beta weights show that individual VSR to the difficult task decreased with increasing RT and cognitive effort costs. Figure 7 shows the corresponding partial regression plots. Finally, we conducted a sensitivity analysis for multiple linear regression using GPower (*α* = 0.01, power = 0.95, *N* = 96, 3 predictors) which resulted in a minimum effect size of 0.25 and a critical *F*-value of 4.00 allowing to interpret the significant effects of our analyses as true positives (Faul et al., 2009).

##### VSR to the difficult task when reward prospect increases as DV

**Fig. 8:**
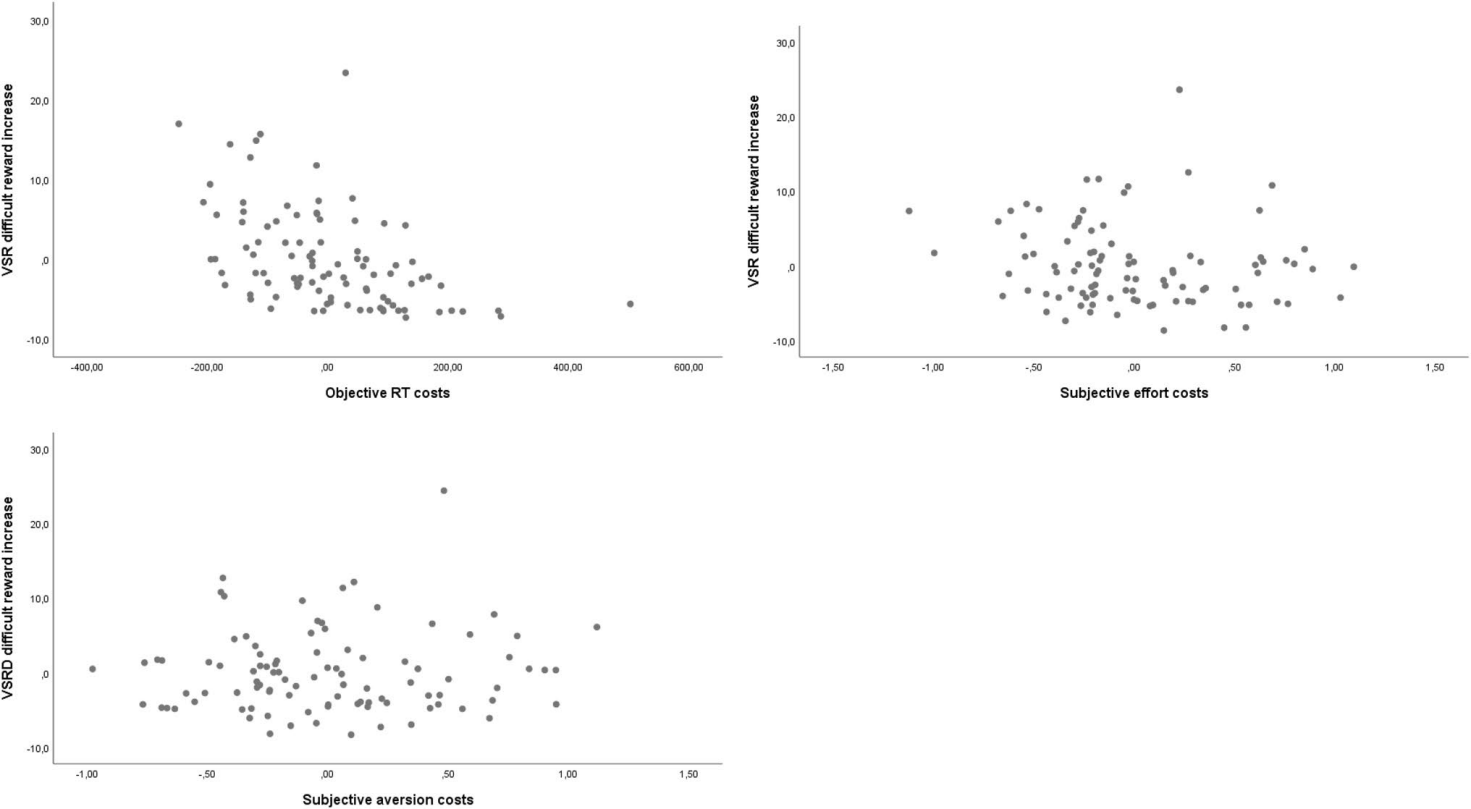
Partial regression plots (Experiment 2) of the VSR to the difficult task when reward prospect increases vs. objective effort costs (upper left panel), vs. subjective effort costs (right panel), and vs. subjective aversion costs (lower panel). *VSR* voluntary switch rate

Again, the regression equation was significant, *F*(2,95) = 12.21, *p* < 0.001. *R*^2^ for the overall model was 0.28 (adjusted *R*^2^ = 0.26), again indicative for a high goodness-of- (Cohen, 1988). Participants’ predicted VSR to the difficult task when reward prospect increases is 11.41–0.024 (RT costs) – 1.86 (subjective effort costs) + 0.76 (subjective aversion costs). However, only the actual RT costs were a significant predictor of the VSR to the difficult task, *β* = − 0.49, *t*(95) = − 5.18, *p* < 0.001, whereas subjective effort cost, *β* = − 0.156, *t*(95) = − 1.46, *p* = 0.14, and the subjective aversion costs were not, *β* = 0.07, *t*(95) = 0.595, *p* = 0.55. The negative beta weights show that individual VSR to the difficult task on reward increase trials decreased with increasing effort costs and non-significantly with increasing subjective effort costs. Figure 8 shows the corresponding partial regression plots.

### Discussion

In Experiment 2, we aimed to further investigate the potential role for subjective effort and aversion costs during voluntary task choice between an easy and a difficult task. With respect to the task switching block, the basic effects from Experiment 1 were perfectly replicated. Likewise, we replicated the association between objective RT costs for the difficult task and the VSR to the difficult task (when reward prospect increased). The lower the actual performance cost for the difficult task of a given participant, the higher the VSR to this task. But we found only weak support for the idea that subjective effort costs measured in an unrelated math task further explain the voluntary choice for the prime task. With respect to the overall VSR, we in fact found a (just) significant effect, suggesting that some general effort avoidance may have biased participants against switching to the difficult task in general, but this effect played no role when reward prospect increased. Finally, we did not find any effect for the subjective aversion costs on voluntary choice for the difficult task.

## General discussion

With the present study, we aimed to investigate two potential sources for the so-called effort paradox (Inzlicht et al., 2018) that describes the phenomenon that people occasionally seem to prefer a more effortful task over a less effortful one. We argued that for some people the allegedly more effortful task in fact is not more effortful and/or that some people simply do not mind the additional effort. That is, humans may decide for a more difficult task either because the more difficult task does not incur higher performance costs (objective costs) or because they do not mind the higher effort costs (subjective costs). To this end, our participants made iterative choices between an easy and a more difficult task in a hybrid task switching paradigm with forced and free choices. We measured the objective RT costs as the difference between the difficult and the easy task. And we used the EDT (Westbrook et al., 2013) to measure the subjective effort costs for the difficult task (Experiment 1) or for a new difficult task and a low-fluency task (Experiment 2). The main findings can be summarized as follows: (1) Overall, participants strongly prefer the easy over the difficult task as indicated by the generally higher VSR to the easy task. (2) In both experiments, a subset of participants sometimes voluntarily chooses the difficult task, especially so when reward prospect increases. (3) This latter effect is predicted by the objective performance cost: the lower these performance costs for an individual, the higher the VSR to the difficult task (when reward prospect increases). (4) We found only marginal support for the idea that the subjective effort costs further modulate voluntary task choice: In Experiment 2, the subjective effort costs were a (just) significant predictor of the overall VSR to the difficult task (but not for the VSR when reward prospect increased). The subjective aversion costs were no reliable predictor.

The VSR data thus perfectly replicate previous findings from Jurczyk et al., (2019). The association found with the objective costs here suggests that the sequential reward effect for the difficult task may underly the same mechanisms as the sequential reward effect in general. As already outlined in the introduction and elsewhere, we argue that the general reward sequence effect (higher VSR when reward prospect increases than when reward remains high) could be explained by a lowering of the updating threshold in working memory in response to the unexpected increase in reward (Cools & D’Esposito, 2011; Dreisbach & Fröber, 2019; Fröber & Dreisbach, 2020). That means, we do not need to assume an additional mechanism to explain the same phenomenon for the VSR to the difficult task because those participants who switch to the more difficult task are those who do not show much of a performance difference between both tasks. Now, one may argue that the RT costs are not cause but rather effect of the higher VSR to the difficult task. In other words, participants show lower objective RT costs *because* they choose the difficult task more often and therefore have more practice with the difficult task. While we cannot rule out this argument in its entirety, we think that this argument would only hold if we had used a “pure” voluntary task switching paradigm. Remember that we used the hybrid task switching paradigm with 50% forced choices. By this we made sure that all participants had to do the difficult task on 25% of all trials. This made sure that all participants received the same amount of practice with the difficult task on forced choices (from which the RT costs were measured). We therefore think that a potential practice effect from the higher VSR may at best have added to the effect but cannot explain it entirely.

To our knowledge, this is the first study that administered the cognitive effort discounting paradigm (COG-ED) to predict choice behavior between tasks of unequal difficulty. In the original study by Westbrook et al. (2013), the authors showed that the paradigm is sensitive to task load in a *n*-back working memory task: subjective effort costs increased with increasing working memory load (*n*), confirming an overall tendency towards effort avoidance. And they also showed that these costs vary between participants depending on cognitive engagement as measured by the Need for Cognition Scale (NCS, Cacioppo et al., 1984) and on age, and cannot be explained by task performance: the subjective effort costs were negatively correlated with the NCS and older participants tended to discount more. This association between subjective effort costs and task difficulty has recently been confirmed for children (Chevalier, 2018) and adolescents (Kramer et al., 2021). However, whether and to what extent these costs are suited to predict effortful behavior has not been investigated. Therefore, at the moment, we can only speculate about the reasons for the only moderate effect found in Experiment 2. One potential reason may be that the effort discounting procedure is not sensitive enough to measure individual differences in effort avoidance independent from the individual performance in the respective task. This might at least explain why we did not find any effect in Experiment 1 where we used the same tasks during voluntary task switching (as the dependent measure) and the EDT (as the predictor). But it might also be true that the bias in favor of the easy task was just too strong leaving not much room for the potential influence of the individual effort costs. In any case, we still think that the EDT is a promising tool to measure the individual willingness to exert effort and therefore deserves further examination.

The results presented here complement recent studies investigating voluntary choice behavior in task switching. For example, Mittelstädt and colleagues (2018a, 2018b) manipulated the stimulus onset asynchrony (SOA) for task repetitions with increasing numbers of direct (voluntary) task repetitions. That is, the stimulus for a potential task switch was made available immediately whereas the stimulus for a potential repetition was only presented after a certain (adaptively increasing) interval. It turned out that participants switched more often when the SOA and thus the costs for waiting approached or even exceeded the costs of switching. This finding converges with ours in that it again shows that participants are sensitive to performance costs and adjust their task choice accordingly. Likewise, the authors repeatedly found a significant correlation between switch costs and the repetition rate (Mittelstädt et al., 2018a, 2018b), again supporting our claim that participants’ choice for a task repetition or shift is—to a large part—determined by economic (RT costs) considerations. Likewise, Mayr and Bell (2006) had shown that switch costs in a block of forced alternating runs (AABB) correlated negatively with the VSR in a then following voluntary task switching block. Our findings extend these observations as we have shown that not only switch costs per se but also the differential performance costs for a given task (and to a lesser degree individual effort avoidance as measured by the EDT) modulate this choice behavior. Given that tasks in everyday life are rarely balanced for difficulty and effort, this finding furthers our understanding of human action selection.

Another aspect we would like to address shortly is the relationship between objective effort costs and performance costs. Critically, the invested effort cannot directly be inferred from performance like the RT difference between the difficult and the easy task alone. After all, performance in a given task is a result of ability, success importance and invested effort, and most presumably a mixture of all three (Wright et al., 2019). That is, to obtain the same result, some participants may have to invest more effort than others (e.g., Smith & Hess, 2015). This reasoning has already been explicated in the motivational intensity theory (Brehm & Self, 1989) which predicts—for known difficulty, as is the case in our experiment—increasing effort investment with increasing task difficulty (until the invested effort is no longer justified by the obtained outcome). Support for this association come from studies, using (sympathetic) cardiovascular reactivity scores as objective measures of effort, showing increasing reactivity with increasing task difficulty (for a review see Richter et al., 2016). Even though performance measures (depending on task difficulty) and cardiovascular measures often correlate (e.g., Richter et al., 2008), this does not necessarily have to be the case.

On a more general level, the obtained results allow to conclude that participants who voluntarily choose the more difficult task may be those who do not pay a high price for the additional effort in terms of actual RT costs. This is an important finding because it shows that simply observing choice behavior without taking individual ability and performance into account can be misleading. That means, our results show that the effort-paradox can in part be explained by the fact that effort is not an objective feature of an action but critically depends on the individual’s capability and motivation. This sounds like a rather obvious statement but in daily life we are often tempted to draw the wrong conclusions from action observation. For example, we tend to judge the neighbor who is going for a morning run every day in all kinds of weather for a highly disciplined person when in fact, running for her might be effortless and discipline is only needed to follow the doctor’s advice for recreation. In other words, whereas action selection can be readily observed, the individually invested costs that are associated with the chosen action are harder to infer. Therefore, what might seem like an either irrational (“why doing more than necessary?”) or disciplined (“always giving your best”) choice might in fact be an economic and thus rational choice for the individual.

In sum, our results suggest that choosing a more difficult task is not primarily a matter of “willpower” (Gailliot & Baumeister, 2007) but that in fact the actual performance costs (or the lack thereof) may contribute a good share to the opportunity costs that arise when choosing one task over the other (Kurzban et al., 2013). We do not mean to rule out that humans do also favor a more challenging task for other reasons than the lack of higher costs (as presented here). Inzlicht and colleagues (2018) gathered convincing evidence that humans may also favor a more difficult task because the invested effort adds value to the product of effort as is exemplified in the so-called IKEA effect (Norton et al., 2012) or because effort itself is intrinsically rewarding (Eisenberger, 1992). Note, however, that the reward prospect in our study was always contingent on performance but *non*-contingent on task choice [as opposed to (Braun & Arrington, 2018)]. That is, the (sequentially changing) reward prospect in our study presumably only changed the meta-control state in favor of a more or less flexible control mode (e.g., Dreisbach & Fröber, 2019; Goschke & Bolte, 2014; Hommel, 2015) and thereby the probability to switch. That is, the reward prospect in our study affected task choice only indirectly as opposed to most studies that use reward to directly motivate a certain task choice (Braun & Arrington, 2018). It will be an interesting endeavor for future research to find ways to investigate potential interactions between these different sources of the effort paradox. For example, it is conceivable that a person with a long learning history that effort is usually rewarded—as it is suggested by the learned industriousness approach—might learn to succeed in all sorts of tasks thereby perpetuating the choices for more challenging tasks because their mastery reduces actual performance costs.

The take home message of the present study is that voluntary task choice can be modulated by (1) the control state (here manipulated via changing reward prospects) and (2) the individual performance costs for the chosen task. An increase in reward prospect increases cognitive flexibility and the general likelihood to switch. The individual task choice follows economic considerations incorporating the individual performance costs for the chosen task.

## Supplementary Information

Below is the link to the electronic supplementary material.

Supplementary file1 (DOCX 29 KB)

## Data Availability

Raw data files associated with this article can be found online under the following link https://epub.uni-regensburg.de/47889/.

## Footnotes

1 We are aware of the phenomenon of asymmetrical switch costs which describe the well documented observation that a switch to the more difficult task can be easier than a switch to the easier task. However, this asymmetry is typically only observed when two tasks that operate on the identical stimulus set are used. Since we will use univalent stimuli (letters and digits), asymmetrical switch costs will not be an issue. That means, the switch to the difficult task will in fact be accompanied by higher costs than a switch to the easy task.

2 Whether the first forced choice trial after a free choice trial constitutes a switch or repetition was necessarily contingent on an individual’s choice. That means that for a participant who voluntarily switches more often to the difficult task it is more likely that an easy task as the first forced choice (in a row) constitutes a task switch. And conversely, for a participant who switches voluntarily more often to the easy task it is more likely that an easy forced choice task is a task repetition. That is, while transition (repeat vs. switch) and task type (easy vs. difficult) were equally distributed on forced choices, the specific factor combination was not. In fact, a participant who voluntarily switched more often to the difficult task was relatively more likely to receive fewer forced switches to the difficult task, and a participant who voluntarily switched less often to the difficult task was relatively more likely to receive more forced switches to the difficult task. This is not a critical confound because, if anything, it would work against our hypothesis: After all, more (and not less) forced switches to stimuli of the difficult task should increase VSR to the difficult task (see Chiu, Fröber & Egner, 2020).

3 Note that we never mentioned the words “difficult” vs. “easy” task in the instructions but always described the tasks by their respective stimuli (digit vs. letter).

4 RTs and error rates on voluntary choices are presented in the supplemental online materials.

5 Exclusion of these participants had no impact on the statistical pattern of results obtained.

6 To account for the slight imbalances within the factor combination for switches and repetitions to the difficult and easy task on forced choices, we also computed this difference score over the factor level combinations (i.e. [meanDifficult_switch_ + meanDifficult_rep_]—[meanEasy_switch_ + meanEasy_rep_]. This difference measure was highly correlated with the respective performance costs based on the raw data (*r* = .94, *p* < .001) and the regression analyses using this RT cost difference as predictor yielded qualitatively the same results.

7 As in Experiment 1, the alternative RT difference based on the respective mean RTs for switches and repetitions to the easy and difficult task were highly correlated with these performance costs (*r* = .95, *p* < .001) and entering this measure into the regression analyses yielded qualitatively the same results.
