# Supplementary material for: The role of objective and subjective effort costs in voluntary task choice
Source: Psychol Res. 2021 Aug 29;86(5):1366–81. doi: 10.1007/s00426-021-01587-2 (PMC9177489; doi:10.1007/s00426-021-01587-2)
Supplement: Supplementary file 1 — Supplementary file1 (DOCX 29 KB) [file 426_2021_1587_MOESM1_ESM.docx]

**Supplemental Online Materials**

**Experiment 1**

**Free-choice trials, RT data.** The same 2 (Difficulty) × 2 (Transition) × 4 (Reward Sequence) repeated measures ANOVA on free choice trials was conducted only on a subset of 23 participants because all other participants had at least one empty design cell (see Table S1 for descriptive statistics). This analysis revealed a significant main effect of task transition, *F*(1, 22) = 8.66, *p* < .01, $\text{η}_{p}^{\text{2}}$ = .28. RTs were slower on switch trials as compared to repeat trials (668 ms vs. 579 ms). Likewise, reward sequence was significant with *F*(3, 22) = 2.94, *p* = .04, $\text{η}_{p}^{\text{2}}$ = .19. Participants again were faster on increase and remain high trials (601 ms, 588 ms) as compared to remain low and decrease trials (641 ms, 664 ms). The factor difficulty was only marginally significant, *F*(1, 22), 3.98, *p* = .059, $\text{η}_{p}^{\text{2}}$= .15. There were no significant interactions (all *p* > .1).

**Free-choice trials, Error data.** The same ANOVA on the error data could be conducted on a subset of 29 participants (note that a participant who attempts to switch to a difficult task, as denoted by a hand switch but a wrong key press, produces an error on this trial but no response time, this explains why the subset of participants for the RT and error analysis on free choice trials is not necessarily the same). Aside from a main effect difficulty, *F*(1, 28) = 23.29, *p* < .01, $\text{η}_{p}^{\text{2}}$ = .45, no other main effect or interaction was significant (all *p* > .2). Participants made more errors on the difficult task than on the easy task (16 % vs. 8 %).

Table S1: Mean RT (ms), mean Error rates (%) and their respective standard error of the mean of free choice trials in Experiment 1. Results should be taken with caution as they are only based on a subset of participants who had no empty design cells (N = 23 for RT, N = 29 for error rates).

| Task | Transition | RewSeq | Mean RT (ms) | SEM | Mean  Error (%) | SEM |
| --- | --- | --- | --- | --- | --- | --- |
| difficult | rep | remain low | 653 | 35 | 16.7 | 2.9 |
|  |  | increase | 593 | 21 | 15.0 | 2.5 |
|  |  | remain high | 589 | 26 | 15.3 | 1.6 |
|  |  | decrease | 676 | 22 | 14.5 | 2.9 |
|  | switch | remain low | 683 | 30 | 16.8 | 3.9 |
|  |  | increase | 677 | 51 | 19.1 | 3.9 |
|  |  | remain high | 675 | 48 | 15.7 | 4.5 |
|  |  | decrease | 693 | 47 | 18.6 | 4.8 |
| easy | rep | remain low | 538 | 14 | 8.1 | 1.0 |
|  |  | increase | 538 | 29 | 10.5 | 1.3 |
|  |  | remain high | 502 | 17 | 9.5 | 1.3 |
|  |  | decrease | 541 | 17 | 6.9 | 1.3 |
|  | switch | remain low | 689 | 57 | 7.6 | 2.7 |
|  |  | increase | 596 | 46 | 11.3 | 2.9 |
|  |  | remain high | 585 | 26 | 6.2 | 1.8 |
|  |  | decrease | 747 | 140 | 5.5 | 1.5 |

**Experiment 2**

**Free-choice trials, RT data.** As for experiment 1, there were again a lot of empty design cells for a given participant whose data therefore were not included in the RT analysis, which is based on 29 participants only. Their mean RTs were entered into a 2 (Difficulty) × 2 (Transition) × 4 (Reward Sequence) ANOVA with repeated measures (see Table S2 for descriptive statistics). The main effects of difficulty, *F*(1, 28) = 32.77, *p* < .01, $\text{η}_{p}^{\text{2}}$ = .54, and task transition, *F*(1, 28) = 30.4, *p* < .01, $\text{η}_{p}^{\text{2}}= .52,$and reward sequence, *F*(3, 28) = 9.98, *p* < .01, $\text{η}_{p}^{\text{2}}= .26$ were significant. Participants were slower on difficult as compared to easy trials (718 ms vs. 602 ms), they were faster when they repeated a task (607 ms vs. 714 ms) and they were faster when reward increased or remained high (605 ms, 628 ms, respectively) than when reward remained low or decreased (722 ms and 685 ms). Moreover, reward sequence interacted significantly with transition, *F*(3, 28) = 5.89, *p* < .01, $\text{η}_{p}^{\text{2}}= .17$. This interaction again confirmed that switch costs are smaller when reward prospect increase than when reward remains high (39 ms vs. 126ms).

**Free-choice trials, Error data.**

The same ANOVA was conducted on a subset of 39 participants. There were significant main effects of difficulty, F(1, 38) = 28.60, p < .01, $\text{η}_{p}^{\text{2}}$ = .42, and of transition, *F*(1, 38) = 6.09, *p* < .02, $\text{η}_{p}^{\text{2}}$ = .14. Participants made more errors on difficult trials (18 % vs. 7 %), and they made more errors on switch trials (15 % vs. 11 %). Both factors interacted significantly, *F*(1, 38) = 5.01, *p* < .05, $\text{η}_{p}^{\text{2}}$ = .12. Participants produced error switch costs in the difficult task (22 % vs. 15 %) but not in the easy task (7% on repetitions and switches alike). No other main effect or interaction was significant (all *p* > .07).

*Table S2*: Mean RT (ms), mean Error rates (%) and their respective standard error of the mean of free choice trials in Experiment 2. Results should be taken with caution as they are only based on a subset of participants who had no empty design cells (N = 29 for RT analysis, N = 39 for error analysis).

| Task | Trans | RewSeq | Mean RT (ms) | SEM | Mean error (%) | SEM |
| --- | --- | --- | --- | --- | --- | --- |
| difficult | rep | remain low | 697 | 31 | 12.2 | 1.7 |
|  |  | increase | 638 | 20 | 16.0 | 1.8 |
|  |  | remain high | 612 | 16 | 15.5 | 1.8 |
|  |  | decrease | 705 | 43 | 15.0 | 1.6 |
|  | switch | remain low | 901 | 856 | 21.5 | 4.5 |
|  |  | increase | 638 | 24 | 22.9 | 4.0 |
|  |  | remain high | 752 | 42 | 25.4 | 5.2 |
|  |  | decrease | 805 | 41 | 17.1 | 3.6 |
| easy | rep | remain low | 567 | 17 | 7.2 | 0.9 |
|  |  | increase | 535 | 13 | 6.5 | 0.9 |
|  |  | remain high | 519 | 11 | 8.9 | 1.3 |
|  |  | decrease | 582 | 17 | 6.1 | 0.8 |
|  | switch | remain low | 724 | 53 | 6.8 | 2.9 |
|  |  | increase | 612 | 25 | 5.2 | 1.6 |
|  |  | remain high | 630 | 33 | 8.9 | 1.9 |
|  |  | decrease | 648 | 30 | 8.4 | 1.9 |
